# Novel *FGFR1* Variants Are Associated with Congenital Scoliosis

**DOI:** 10.3390/genes12081126

**Published:** 2021-07-24

**Authors:** Shengru Wang, Xiran Chai, Zihui Yan, Sen Zhao, Yang Yang, Xiaoxin Li, Yuchen Niu, Guanfeng Lin, Zhe Su, Zhihong Wu, Terry Jianguo Zhang, Nan Wu

**Affiliations:** 1 Department of Orthopedic Surgery, State Key Laboratory of Complex Severe and Rare Diseases, Peking Union Medical College Hospital, Peking Union Medical College and Chinese Academy of Medical Sciences, Beijing 100730, China; wangshengru@foxmail.com (S.W.); chaixiran1124@163.com (X.C.); yanzihui1127@126.com (Z.Y.); zhaosen830@163.com (S.Z.); kaido137@hotmail.com (Y.Y.); richard_lingf@foxmail.com (G.L.); suesu0092@126.com (Z.S.); orthoscience@126.com (Z.W.); dr.wunan@pumch.cn (N.W.); 2 Key Laboratory of Big Data for Spinal Deformities, Chinese Academy of Medical Sciences, Beijing 100730, China; ldx217@yeah.net (X.L.); nhtniuyuchen@126.com (Y.N.); 3 Graduate School of Peking Union Medical College, Beijing 100005, China; 4 Beijing Key Laboratory for Genetic Research of Skeletal Deformity, Beijing 100730, China; 5 Medical Research Center, State Key Laboratory of Complex Severe and Rare Diseases, Peking Union Medical College Hospital, Peking Union Medical College and Chinese Academy of Medical Sciences, Beijing 100000, China

**Keywords:** *FGFR1* (Fibroblast growth factor receptor 1), genetic variations, congenital scoliosis

## Abstract

*FGFR1* encodes a transmembrane cytokine receptor, which is involved in the early development of the human embryo and plays an important role in gastrulation, organ specification and patterning of various tissues. Pathogenic *FGFR1* variants have been associated with Kallmann syndrome and hypogonadotropic hypogonadism. In our congenital scoliosis (CS) patient series of 424 sporadic CS patients under the framework of the Deciphering disorders Involving Scoliosis and COmorbidities (DISCO) study, we identified four unrelated patients harboring *FGFR1* variants, including one frameshift and three missense variants. These variants were predicted to be deleterious by in silico prediction and conservation analysis. Signaling activities and expression levels of the mutated protein were evaluated in vitro and compared to that of the wild type (WT) *FGFR1*. As a result, the overall protein expressions of c.2334dupC, c.2339T>C and c.1261A>G were reduced to 43.9%, 63.4% and 77.4%, respectively. By the reporter gene assay, we observed significantly reduced activity for c.2334dupC, c.2339T>C and c.1261A>G, indicating the diminished FGFR1 signaling pathway. In conclusion, *FGFR1* variants identified in our patients led to only mild disruption to protein function, caused milder skeletal and cardiac phenotypes than those reported previously.

## 1. Introduction

The Fibroblast growth factor receptor 1 (*FGFR1*) gene encodes a transmembrane cytokine receptor, which comprises an extracellular region of three immunoglobulin-like domains (D1, D2 and D3), a transmembrane helix and a cytoplasmic tyrosine kinase domain [1]. Although different isoforms have different tissue expression and varied affinity to FGFs, *FGFR1-IIIc*, spliced through the use of exon 8B, is the predominant isoform that carries out most of the functions of the *FGFR1* gene [2].

The downstream signaling of *FGFR1* is activated by the dimerization and activation of the receptor and autophosphorylation of the tyrosine kinase domains. These downstream signaling pathways include the mitogen activated protein kinases (MAPK), the phosphatidylinositide 3 kinase/AKT (PI3K/AKT) and the phospholipase C γ (PLC) [1,3]. *FGFR1*-related signaling pathways are involved in the early development of the human embryo, and thus play an important role in gastrulation, organ specification and patterning of many tissues [4].

Many *FGFR1* mutations have been identified in both Kallmann syndrome and isolated hypogonadotropic hypogonadism (IHH) [5,6,7,8,9]. *FGFR1* loss-of-function mutations were also reported to be found in Kallmann syndrome patients with skeletal phenotypes, including oligodactyly, hemivertebrae and butterfly vertebrae [10] and *FGFR1* signaling was reported to be important for different stages of osteoblast maturation [11]. Mice models with *FGFR1* variants presented various skeletal phenotypes, especially vertebral malformation from cervical vertebrae to lumbar vertebrae, making *FGFR1* a candidate gene for congenital scoliosis [12]. However, whether *FGFR1* is associated with vertebral malformations in human remains unknown.

In this study, we analyzed variants of *FGFR1* identified in a cohort of congenital scoliosis (CS) and performed in vitro experiments to determine the effects of these variants on the protein function.

## 2. Materials and Methods

### 2.1. Human Subjects

A total of 424 sporadic Han Chinese probands who received a diagnosis of congenital scoliosis (CS) were consecutively collected into the cohort between 2009 and 2018 at Peking Union Medical College Hospital (PUMCH) under the the framework of the Deciphering disorders Involving Scoliosis and COmorbidities (DISCO, http://discostudy.org/, accessed on 15 March 2021) study. Demographic information, physical examination results, clinical symptoms on presentation, and a detailed medical history were obtained from each proband. Clinical diagnoses were confirmed by radiology imaging. Blood was obtained from all the probands and whole exome sequencing (WES) was performed.

A total of 942 Han Chinese individuals without evidence of congenital scoliosis or other congenital malformations from the DISCO project served as in-house controls. All in-house controls provided their blood for DNA analysis and signed written informed consent.

### 2.2. Bioinformatic Analysis and Mutation Interpretation

WES data processing was performed using the Peking Union Medical college hospital Pipeline (PUMP) [13,14] developed in-house. Computational prediction tools (Genomic Evolutionary Rate Profiling [GERP] [15], Combined Annotation Dependent Depletion [CADD PHRED-score, GRCh37-v1.6] [16], Sorting Intolerant Form Tolerant [SIFT] [17], Polyphen-2 [18], and MutationTaster [19]) were used to predict the conservation and pathogenicity of candidate variants. All variants were compared against population genomic databases such as the 1000 Genomes Project (http://www.internationalgenome.org/, accessed on 15 March 2021), the NHLBI GO Exome Sequencing Project (ESP) Exome Variant Server (http://evs.gs.washington.edu/EVS/, accessed on 15 March 2021) and the genome Aggregation Database (gnomAD, http://gnomad.broadinstitute.org/, accessed on 15 March 2021).

Candidate variants in *FGFR1* were extracted and filtered using the following criteria:

(1) Truncating (nonsense, frameshift, splice acceptor/donor) variants or missense variants/inframe indels with a CADD score ≥ 20;
(2) Absent from population genomic databases listed above.

### 2.3. Site-Directed Mutagenesis

Plasmids of pcDNA3.1+ with N-terminal myc-tagged WT and mutant *FGFR1c* cDNA (NM_023110.2) were acquired from Beijing Hitrobio Biotechnology. The mutant constructs were sequenced on both strands to verify nucleotide changes.

### 2.4. Receptor Expression and Maturation Studies

#### 2.4.1. Endoglycosidase Digestion

Endoglycosidase assays were performed as previously published [8]. In brief, COS-7 cells (Cell Resource Center, Peking Union Medical College, Beijing, China) with 60–70% confluence were transiently transfected with 300 ng of plasmid containing myc-tagged WT or mutated *FGFR1* cDNA in 6-well plates using Lipofectamine 3000 reagent (Thermo Fisher Scientific, Waltham, MA, USA). Forty-eight hours post transfection, cells were washed with phosphate-buffered saline (PBS), and then, lysed with 100 μL of RIPA buffer (Thermo Fisher Scientific, Waltham, MA, USA) containing 1× protease inhibitor (Solarbio, Beijing, China). For deglycosylation analysis, all protein lysates were diluted to 10 μg/μL, and 9 μL of diluted lysate (90 μg of total protein) was subjected to PNGasef and EndoH digestion according to the manufacturer’s recommendations (New England Biolab, Ipswich, MA, USA).

#### 2.4.2. Western Analysis

Untreated or endoglycosidase-treated samples were resolved on gels under reducing conditions and then, subjected to Western analysis using an anti-myc primary antibody (clone 4A6, 1:1000, Upstate Biotechnology, Inc., Lake Placid, NY, USA) and a goat anti-mouse horseradish peroxidase-conjugated secondary antibody (1:5000, Bioss, Edinburgh, UK). Immunoreactivity was visualized using Western Lighting chemiluminescence reagent (Beyotime, Wuhan, China). To control for equal loading, blots were stripped using Restore Western Blot Stripping Buffer (Applygen Technologies, Beijing, China) and reprobed with horseradish peroxidase-conjugated anti-β-actin antibody (1:5000, Proteintech, Rosemont, IL, USA). FGFR1 and β-actin immunoreactivity were quantified by densitometry using an automatic chemiluminescence imaging system (Tanon, Shanghai, China). Overall expression levels of WT and mutant receptors were determined from the PNGase-treated samples and were normalized to their respective β-actin levels. The ratio between mutant and WT was reported. For receptor maturation studies, the upper (mature) and lower (immature) band densities were determined individually from the EndoH-treated samples, and the percent of mature fraction (maturation level) was calculated as overall protein divided by matured protein. The maturation levels of four variants were compared with the WT group, i.e., maturation ratio. Endoglycosidase and Western experiments were repeated three times.

#### 2.4.3. FGF Reporter Gene Assay

The activation of downstream signaling pathways by wild type and mutated *FGFR1* constructs was interrogated using the luciferase-based reporter assay; the osteocalcin FGF response element (OCFRE) reporter reports the activity of the MAPK pathway downstream of FRS2α signaling [9]. In detail, L6 myoblasts (Cell Resource Center, Peking Union Medical College, Beijing, China), which are largely devoid of endogenous FGFRs and FGFs, were maintained in DMEM-H containing penicillin (100 U/L), streptomycin (100 ug/L), and 10% fetal calf serum. Transient transfections were performed at 60–70% cell confluency in 24-well plates with 300 ng of plasmid containing WT or mutant *FGFR1* cDNA, 400 ng of osteocalcin FGF response element-pGL3 plasmid, and 10 ng of pRL plasmid using Lipofectamine 3000 reagent (Thermo Fisher Scientific, Waltham, MA, USA). After 24 h of serum starvation, cells were treated for 16 h with FGF18 (10-8 M) in DMEM-H containing 0.1% BSA. The cells were lysed with passive lysis buffer (Promega, Madison, WI, USA), and assayed for luciferase activity using a Promega luciferase assay system. Experiments were performed in triplicate and repeated at least three times. Results of each experiment were normalized to the WT and the mean values of three experiments were calculated.

#### 2.4.4. Statistical Analyses

The frequency of candidate variants of *FGFR1* was compared between the control group and the CS group using the Fisher Exact Test. Luciferase activities and overall expression levels were normalized to WT (set as 100%) and mean values of mutant versus WT from all three experiments were compared using one-way ANOVA and Dunnett’s multiple comparisons test. All charts were drawn and analyzed using GraphPad Prism 7 and *p* < 0.05 was considered significant for all analyses.

## 3. Results

### 3.1. Mutation and Phenotype Analyses

In the 424 sporadic CS patients, 79 patients (18.6%) were found to have a molecular diagnosis by pathogenic genetic variants, as previously reported [13]. From the probands who remained undiagnosed, four likely deleterious heterozygous variants of *FGFR1*, including one frameshift variant and three rare missense variants (c.2334dupC; c.2339T>C; c.1107G>A; c.1261A>G), were identified (Table 1), presenting a significant mutational burden as compared with the in-house controls (one candidate variant in 942 control individuals, *p* = 0.035, Fisher Exact Test). The authenticity of all variants was validated by manual review of BAM files using the Integrative Genomics Viewer (http://igv.org, accessed on 15 March 2021).

**Figure 1:**
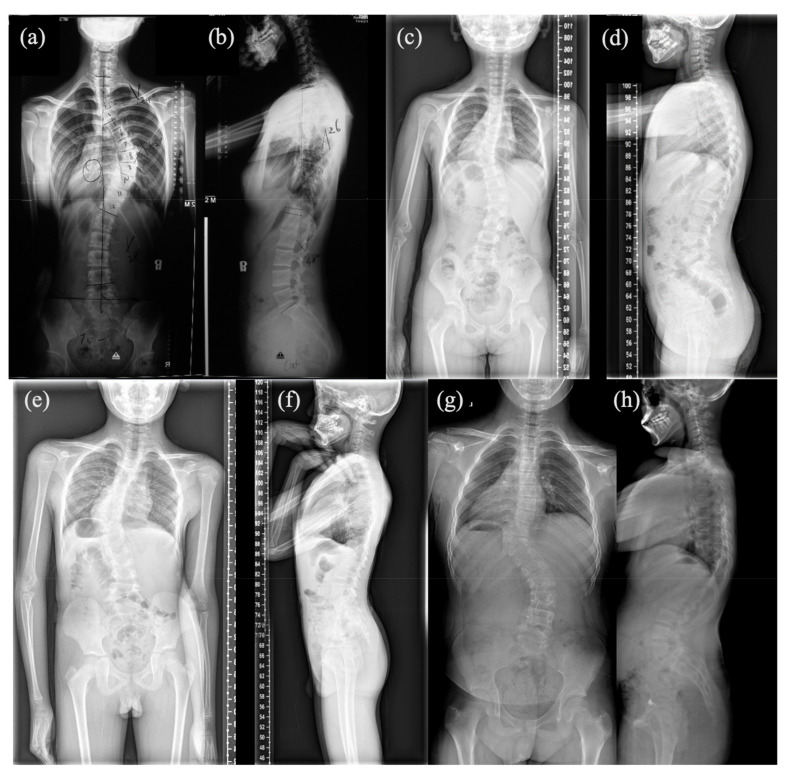
Anteroposterior and lateral spinal X-ray of four patients: (**a**,**b**) Anteroposterior and lateral spinal X-ray of patient #1; (**c**,**d**) Anteroposterior and lateral spinal X-ray of patient #2; (**e**,**f**) Anteroposterior and lateral spinal X-ray of patient #3; (**g**,**h**) Anteroposterior and lateral spinal X-ray of patient #4.

**Figure 2:**
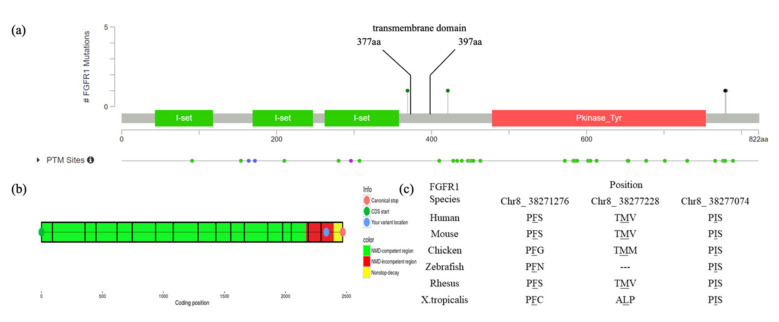
Mapping and conservation analysis of four variants: (**a**) Mapping of four *FGFR1* variants revealed that c.2334dupC and c.2339T>C are located in the intracellular region and post-translational phosphorylation sites of the FGFR1 protein, whereas c.1261A>G is located in the transmembrane region and close to the post-translational phosphorylation site; (**b**) The result of NMD prediction of c.2334dupC showed that it is located in the NMD-incompetent region; (**c**) Mutation loci of the three missense variants (c.2339T>C, c.1261A>G and c.1107G>A) are highly conservative across different species.

Patient #1 is a 13-year-old female with T6-10 segmentation defect (Figure 1a,b), fused left 9-10 ribs and mitral valve prolapse. She has a heterozygous duplication of nucleotide 2334 (c.2334dupC; p.Ser779GlnfsTer21). The variant was mapped in the intracellular region and post-translational phosphorylation site of the FGFR1 protein (Figure 2a) and was predicted by the NMD (nonsense-mediated decay) Prediction Tool (https://nmdpredictions.shinyapps.io/, accessed on 15 March 2021) to be located in the NMD-incompetent region (Figure 2b), suggesting that the variant is unlikely to cause nonsense-mediated decay. The variant was not found in population genomic databases, such as 1000G, ESP6500 and gnomAD. The CADD PHRED score of this variant is 32, indicating the deleteriousness of this variant.

Patient #2, a 4-year-old female, has T9 hemivertebrae, T8 butterfly vertebrae, and three ribs absent (Figure 1c,d). She has an *FGFR1* missense variant c.2339T>C (p.Phe780Ser). This variant was also mapped in the intracellular region and post-translational phosphorylation sites of the FGFR1 protein (Figure 2a). It was not found in most population databases, such as 1000 G, gnomAD and ESP6500. The variant was highly conservative across different vertebral species (Figure 2c). In silico prediction had contradictory results (tolerant or benign for SIFT, pathogenic for MutationTaster, Polyphen2, LRT and CADD PHRED-score).

Patient #3 is a male newborn affected with a T10 hemivertebrae (Figure 1e,f) with a missense variant c.1107G>A (p.Met369Ile). It is a novel mutation according to all population databases. In silico predictions were tolerant or benign for SIFT and Polyphen2, but deleterious for MutationTaster, LRT and CADD PHRED score.

Patient #4 is a male newborn who presents T10 hemivertebrae (Figure 1g,h). This patient has a novel missense variant (c.1261A>G; p.Ile421Val), which was mapped in the transmembrane region and close to the post-translational phosphorylation site of FGFR1 protein (Figure 2a). This variant is highly conservative among different vertebral species (Figure 2c). It was predicted to be deleterious by SIFT, MutationTaster, LRT and CADD PHRED score.

### 3.2. Functional Characterization of FGFR1 Variants

#### 3.2.1. Western Analysis

**Figure 3:**
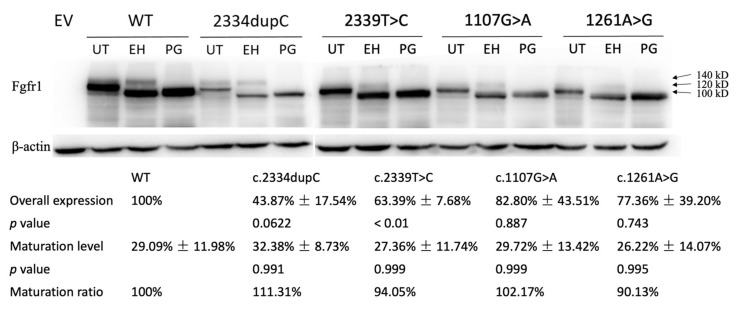
Western blot analysis of COS-7 cells transiently transfected with WT or mutant *FGFR1* constructs reveal diminished protein expression levels of c.2334dupC, c.2339T>C, c.1107G>A and c.1261A>G. Overall expression was significantly decreased in all four variants, especially in c.2334dupC. No difference in protein maturation process was detected using a receptor deglycosylation. EV = empty vector, WT = wild type, UT = untreated, EH = EndoH-treated, PG = PNGase-f-treated.

To identify the influences of these four variants on the function of the FGFR1 protein, we evaluated overall protein expression and maturation of the different *FGFR1* variants compared to WT. Endoglycosidase digestion and Western blotting analysis showed two immunoreactive-specific bands for WT FGFR1 at 140 and 120 kDa, corresponding to a differently N-glycosylated receptor. These two bands were reduced to a single lower molecular weight band following peptide N-glycosidase (PNGase) digestion to remove all types of N-linked carbohydrate chains. Treatment with endoglycosidase H (EndoH), which only removes high mannose N-linked sugars, merely affects the immature form (120 kDa), leaving the fully glycosylated mature form (140 kDa) intact. Thus, maturation rate can be calculated by dividing the band of 140 kDa from EndoH-treated samples into the band of 100 kDa. Overall expression level was quantified by measuring bands from PNGase-treated samples and normalized to the WT group (set as 100%). The overall expression of the frameshift variant was decreased to 43.9% compared with that of WT (*p* = 0.06), and those of three missense variants were reduced to 63.4% (*p* < 0.01), 82.8% (*p* = 0.887), and 77.4% (*p* = 0.743), respectively (Figure 3). As for maturation analysis, densitometric analysis revealed that 29.1% of the WT FGFR1 protein was expressed as a mature form (Figure 3). Consistent with our mapping analysis predicting that all four variants are not localized in the FGFR1 functional ectodomain, these mutant receptors showed no difference in the level of protein maturation, compared to WT (Figure 3).

#### 3.2.2. FGF Reporter Gene Assay

**Figure 4:**
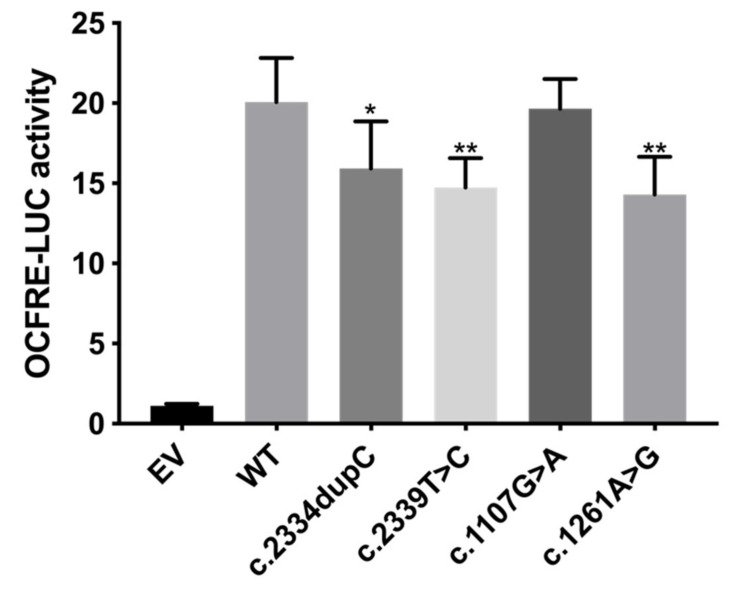
FGF reporter gene assay showing reduced signaling capacity of c.2334dupC, c.2339T>C and c.1261A>G. L6 myoblasts were transiently transfected with OCFRE-luciferase reporter together with wild type (WT) or mutant constructs and then treated with 10-8M FGF18. The average receptor signaling capacities of c.2334dupC, c.2339T>C and c.1261A>G were reduced by 20.7%, 26.6% and 28.8%, respectively. EV = empty vector. * *p* value < 0.05, ** *p* value < 0.01.

To assess the influence of the four *FGFR1* variants on the receptor functionality, we first used the FGF-responsive reporter osteocalcin FGF response element-luciferase in L6 myoblasts, which acts downstream of the MAPK pathway (Figure 4). FGF18 is included in the FGF8 subfamily, which is expressed during somitogenesis and is essential for the morphogenesis of many tissues. In the *FGF18* knockout mice model, skeletal phenotypes have been detected [20], indicating an important role of FGF18 signaling in skeletal development. Previously, FGF18 was found to be expressed in and required for osteogenesis and chondrogenesis [21,22,23,24,25]. Compared to WT FGFR1, the receptor signaling capacity of the truncating variant (c.2334dupC) was reduced by 20.7% (*p* < 0.05, Figure 4). The responses of missense variants (c.2339T>C, c.1261A>G) were also significantly reduced by 26.6% and 28.8%, respectively (*p* < 0.01, Figure 4). These results indicated the diminished signaling pathway of FGFR1 activated by FGF18.

## 4. Discussion

In this study, we identified four pathogenic variants, namely one frameshift and three missense variations in patients with congenital scoliosis. The frameshift variant, c.2334dupC, found in a patient with vertebral segmentation defects and mitral valve prolapse, was the first *FGFR1* variant to be associated with spinal malformations and heart defects. Previous mouse models with *FGFR1* mutations were found to have malformations in both vertebrae and the heart [12], suggesting that *FGFR1* variants were associated with skeletal and cardiac abnormalities. Functional studies of this frameshift variant showed that this variant decreases overall protein expression compared with that of WT with a trend to significance but left protein maturation intact (Figure 3). The decreased overall protein expression of this variant might contribute to the diminished luciferase activity, suggesting a diminished signaling function induced by this variant (Figure 4). As the frameshift variant was located in the NMD-incompetent region, we proposed that this truncating variant did not lead to nonsense-mediated mRNA decay but only mildly affected the protein expression, and thus, merely resulted in mild skeletal and cardiac phenotypes.

As for the three missense variants, all of them were predicted to be deleterious by MutationTaster, LRT and CADD, but had different predictions by SIFT and Polyphen2. Two missense variants (c.2339T>C and c.1261A>G) were highly conservative across a wide range of vertebral species, suggesting them to be deleterious variants. Functional studies revealed that all missense variants had reduced overall protein expression, but only the decrease in c.2339T>C was statistically significant (Figure 3). Further luciferase assay indicated significantly reduced luciferase reporter activities (c.2339T>C and c.1261A>G), and thus, had diminished signaling functions (Figure 4). As the OCFRE reporter used in luciferase assays reports the activity of the MAPK pathway downstream of FRS2α signaling, we can conclude that c.2334dupC, c.2339T>C and c.1261A>G diminish the MAPK pathway.

Western blotting and maturation assay of the missense variant (c.1107G>A) showed a slightly decreased overall protein expression and normal maturation level. However, luciferase assay indicated that this variant has similar luciferase activity compared to WT, suggesting a normal effect on downstream signaling of this variant. As most proteins are redundant regarding their expression level, a minor decrease in expression level might not impact normal function. The missense variant c.1107G>A has an 82.8% expression level and a normal maturation ratio and thus, the matured protein of c.1107G>A is decreased to 84.6% compared to WT (82.8% times 102.17%), while matured protein of the other three variants is decreased to 48.8% for c.2334dupC, 59.6% for c.2339T>A and 69.7% for c.1261A>G compared to WT. Therefore, we proposed that matured protein with less than 70%~80% of WT could not be compensated by the redundant expression and might lead to the diminished signaling function indicated by the luciferase assay.

Furthermore, these three hypomorphic variants, including c.2334dupC, c.2339T>C and c.1261A>G, were mapped around post-translational modification sites and may affect protein phosphorylation, which plays an important role in normal protein function. Ying et al. [26] reported a patient with cryptorchidism, micropenis, strabismus, and hypopsia, who was diagnosed with nIHH. The patient had a de novo mutation in *FGFR1* (c.2008G>A), which induced a post-translational modification defect, including defective glycosylation and impaired trans-autophosphorylation. This study revealed the significance of post-translational modification of FGFR1. Based on in silico analysis and functional study results, we believe these three hypomorphic variants (c.2334dupC, c.2339T>C and c.1261A>G) of *FGFR1* may be associated with spinal defects in our patients. As for patient #3 with c.1107G>A, we propose that his skeletal defects are caused by other unknown genetic or environmental factors.

Pathogenic loss-of-function variants of the *FGFR1* gene were reported to be involved in patients with Kallmann Syndrome, including hypogonadotropic hypogonadism and anosmia [5,10,27], and isolated HH [6,7,8,9]. Patients with *FGFR1* mutations also presented with skeletal phenotypes [7,9,10], including oligodactyly on both feet, fusion of metacarpal bones, hemivertebrae, butterfly vertebrae and split hand/foot malformation.

In our cohort, a broad range of skeletal phenotypes were observed, as one patient had failure of segmentation, one patient had mixed defects and two patients had failure of formation. This is consistent with previous studies of *FGFR1* pathogenic variants, in which patients with HH can present a varied spectrum of reproductive phenotypes and non-reproductive phenotypes [8,10]. Furthermore, different patients carrying identical *FGFR1* mutations were observed to exhibit largely variable expressivity of reproductive phenotypes [8]. *FGFR1* signaling is involved in the determination of mesodermal cell fates and regional patterning of the mesoderm during gastrulation [28], and thus, affects organ specification. For the *FGFR1* signaling pathway, different organ systems respond to ligand binding with discrepant patterns [29], and several distinct downstream pathways, such as Erk1/2, Frs2, Crk proteins and Plcγ, are involved [30]. Given the broad function of *FGFR1* in embryo development, wide crosslink with other signaling pathways, tissue-specific response patterns and different downstream pathways, it is reasonable that patients with *FGFR1* mutations can present distinct phenotypes affecting different organ systems. However, the detailed mechanisms through which *FGFR1* mutations lead to different diseases need to be further studied and clarified.

In previous studies, patients with different FGFR1 domains affected have been revealed to present different phenotype spectra. The variants found in these patients all impair the functional domain of FGFR1 protein, including exon 1U, which is located around multiple transcription factor-binding sites, the FRS2α-binding domain and the tyrosine kinase domain [7,9]. Compared to these studies, patients in our cohort only presented mild spine and heart defects. As none of our variants were located in the functional region of the FGFR1 protein or led to severe damage to protein structure, we hypothesized that mild variants in our patients can only result in mild phenotypes compared with those in previous studies.

## 5. Conclusions

In conclusion, we found four *FGFR1* variants in our CS cohorts—one frameshift variant (c.2334dupC) and three missense variants (c.2339T>C; c.1107G>A; c.1261A>G). Functional studies revealed diminished signaling function and reduced protein expression in three of them (c.2334dupC; c.2339T>C; c.1261A>G). These variants in our patients only caused mild damage to the protein expression, and thus, resulted in mild skeletal and cardiac phenotypes, compared to those in previous studies.

## Figures and Tables

**Table 1:** Demographic, phenotypic and variant information of four patients in our series. All variants’ nomenclatures were based on the *FGFR1* transcript NM_023110.2. All positions were aligned to GRCh37/hg19.

	Patient #1	Patient #2	Patient #3	Patient #4
Sex	Female	Female	Male	Male
Age of onset	11	4	0	1
CS type	Failure of segmentation	Mixed defects	Failure of formation	Failure of formation
Vertebral malformation	T6-T10 Spine fusion	T9 Hemivertebrae, T8 Butterfly vertebrae	T10 Hemivertebrae	T10 Hemivertebrae
Associated anomalies	Mitral valve prolapse; Fusion of 9th and 10th ribs	9th, 10th and 12th ribs absent	None	None
Variant nomenclature	c.2334dupC(p.Ser779GlnfsTer21)	c.2339T>C(p.Phe780Ser)	c.1107G>A(p.Met369Ile)	c.1261A>G(p.Ile421Val)
Mutation type	Frameshift	Missense	Missense	Missense
Position	Chr8_38271280	Chr8_38271276	Chr8_38277228	Chr8_38277074
1000G_ASN_AF	0	0	0	0
gnomAD_EAS_AF	0	0	0	0
ESP6500_AF	0	0	0	0
MutationTaster	NA	1	0.999	1
SIFT	NA	0.53	0.25	0.02
Polyphen2	NA	0.948	0.174	0.481
LRT	NA	0	0	0
CADD PHRED-score	32	24.2	22.8	22.0

AF, allele frequency; pLI, probability of loss-of function intolerance; Ref, reference.

## Data Availability

Data are available upon reasonable request. The datasets analyzed during the current study are available from the corresponding author on reasonable request.
